# Optimal Design of Ceramic Based Hip Implant Composites Using Hybrid AHP-MOORA Approach

**DOI:** 10.3390/ma15113800

**Published:** 2022-05-26

**Authors:** Tej Singh, Chandramani Goswami, Amar Patnaik, László Lendvai

**Affiliations:** 1 Savaria Institute of Technology, Faculty of Informatics, Eötvös Loránd University, 9700 Szombathely, Hungary; sht@inf.elte.hu; 2 Department of Mechanical Engineering, Arya College of Engineering and Information Technology, Jaipur 302028, India; chandramani.goswami10@gmail.com; 3 Department of Mechanical Engineering, Malaviya National Institute of Technology, Jaipur 302017, India; apatnaik.mech@mnit.ac.in; 4 Department of Materials Science and Engineering, Széchenyi István University, 9026 Gyor, Hungary

**Keywords:** hip implant composites, ceramic, physicomechanical, AHP-MOORA, optimisation

## Abstract

Designing excellent hip implant composite material with optimal physical, mechanical and wear properties is challenging. Improper hip implant composite design may result in a premature component and product failure. Therefore, a hybrid decision-making tool was proposed to select the optimal hip implant composite according to several criteria that are probably conflicting. In varying weight proportions, a series of hip implant composite materials containing different ceramics (magnesium oxide, zirconium oxide, chromium oxide, silicon nitride and aluminium oxide) were fabricated and evaluated for wear and physicomechanical properties. The density, void content, hardness, indentation depth, elastic modulus, compressive strength, wear, and fracture toughness values were used to rank the hip implant composites. It was found that the density and void content of the biocomposites remain in the range of 3.920–4.307 g/cm^3^ and 0.0021–0.0089%, respectively. The composite without zirconium oxide exhibits the lowest density (3.920 g/cm^3^), while the void content remains lowest for the composite having no chromium oxide content. The highest values of hardness (28.81 GPa), elastic modulus (291 GPa) and fracture toughness (11.97 MPa.m^1/2^) with the lowest wear (0.0071 mm^3^/million cycles) were exhibited by the composites having 83 wt.% of aluminium oxide and 10 wt.% of zirconium oxide. The experimental results are compositional dependent and without any visible trend. As a result, selecting the best composites among a group of composite alternatives becomes challenging. Therefore, a hybrid AHP-MOORA based multi-criteria decision-making approach was adopted to choose the best composite alternative. The AHP (analytic hierarchy process) was used to calculate the criteria weight, and MOORA (multiple objective optimisation on the basis of ratio analysis) was used to rank the composites. The outcomes revealed that the hip implant composite with 83 wt.% aluminium oxide, 10 wt.% zirconium oxide, 5 wt.% silicon nitride, 3 wt.% magnesium oxide, and 1.5 wt.% chromium oxide had the best qualities. Finally, sensitivity analysis was conducted to determine the ranking’s robustness and stability concerning the criterion weight.

## 1. Introduction

Over one million knee and hip replacements are performed each year, providing patients with improved mobility, pain relief, and personal satisfaction. According to reports, approximately 3.48 million knee replacements will be necessary by 2030, representing a 673 percent increase over the current number of treatments [1,2]. Total hip replacement is one of the best orthopaedic surgeries and has been widely used for re-establishing mobility to hip joints for decades; however, design-related complexities remain a concerning reason for implant failure [3,4]. Today, the main challenges faced by the designers are selecting the proper raw materials in the right amounts, and the design of hip implant composites with excellent mechanical properties with higher wear resistance and biocompatibility [5,6]. For achieving these properties, various material combinations of metals, polymers and ceramics are reported for hip implant applications [7]. In particular, ceramic-on-ceramic-based hip implant composites have received much attention and are becoming increasingly important because of their unique properties, such as higher fracture toughness and wear resistance with excellent chemical stability compared to metal and polymer-based hip implant composites [8].

In 1970, aluminium oxide was the first ceramic material utilised in complete hip arthroplasty [9,10]. However, aluminium oxide leads to catastrophic failure due to its brittle nature [11]. The failure risk can be avoided by introducing zirconium oxide into an aluminium oxide matrix, resulting in a hip implant composite material with higher fracture toughness and reduced wear resistance and hardness [12,13]. To compensate for the hardness and wear resistance loss induced by the presence of zirconia, a tiny proportion of chromium oxide was added [14]. Magnesium oxide was reported to prevent the increase of aluminium oxide grain size during sintering [15]. A homogenous and denser microstructure can be obtained with magnesium oxide, which helps impart strength with increased wear resistance [16]. Zhang et al. [17] studied the influence of magnesium oxide on the mechanical properties and chemical stability of Ce-TZP/Al_2_O_3_ based artificial joint materials. They found that with 0.2 wt.% magnesium oxide content, the various mechanical properties like flexural strength, hardness, and fracture toughness are the best and that it may be employed as a wear-resistant joint replacement material. Several researchers believe silicon nitride could be utilised in hip implant applications due to its higher chemical stability, and favourable mechanical and wear qualities [18]. 

Ceramic-based hip implant composites are gaining popularity due to their superior properties of mechanical (elastic modulus hardness, fracture toughness), high wear and corrosion resistance, and excellent biochemical stability and biocompatibility [10,11,12,13,14,15,16,17,18]. According to the literature, the hardness and strength of ceramic materials such as stabilized zirconium oxide reduced dramatically in water-containing conditions [19]. According to the research, the tetragonal to monoclinic transformation of zirconium oxide is the primary cause of ageing, leading to micro-cracks exacerbated in water vapours [19,20]. The phase transformation further promotes surface roughening, and grain pull out may result in the harmful effect of implanted components [21,22]. When a composite is embedded in the human body, it is well known that it causes several biological reactions. The poor biocompatibility of the implanted materials may result in bacterial infections along with the interface of bone and implanted material, leading to replacements or re-surgeries of the implants [18]. Furthermore, the inserted implants emit ions due to their repeated interactions with the surrounding environment, resulting in a decrease in the performance of the implanted component [23,24,25,26]. It is worth noting that the biochemical stability and biocompatibility of the produced ceramic composites have not been investigated in this current paper, although we understand that these properties are of great importance for end-user applications.

The ultimate performance of the intended hip implant composite depends upon the type and size of the used ingredients with appropriate manufacturing conditions. Hence, the proper attention is required to be paid to the compositional design [27,28]. Moreover, the developed hip implant composites have their own performance implications for the evaluated wear and physicomechanical properties [29]. As a result, selecting the appropriate composition with the desired sound qualities becomes a challenging task that can be accomplished using multi-criteria decision-making (MCDM) tools [30]. These are statistical methods used to tackle complex decision-making problems utilising a finite number of qualities and options [31]. MCDM approaches include AHP (analytic hierarchy process), MOORA (multiple objective optimisation on the basis of ratio analysis), TOPSIS (a technique for the ordering of preference by similarity to ideal solution), VIKOR (vise kriterijumska optimizacija kompromisno resenje), MABAC (multi-attributive border approximation area comparison), SAW (simple additive weighting), MEW (multiplicative exponent weighting), and COPRAS (complex proportional assessment) have been effectively deployed to tackle a variety of decision-making problems [32,33,34,35,36,37]. Among them, MOORA and AHP are very popular. MOORA method is straightforward to understand and implement as it requires significantly fewer computations. The AHP technique, on the other hand, was utilised to determine the relative importance or weight of the criterion employed in various ranking procedures. These approaches are appropriate for decision-making problems and have been successfully implemented in many fields [38,39]. Therefore, in this paper, after manufacturing a series of hip implant composites with varying content of different ceramics and assessing their physical, mechanical, and wear properties, a hybrid AHP-MOORA methodology is proposed for ranking. The main objective is to choose an ideal hip implant composite with the MOORA method, which AHP strengthens for the estimation of criterion weight.

## 2. Experimental Methodology

### 2.1. Materials and Hip Implant Composite Fabrication

Magnesium oxide (MgO), zirconium oxide (ZrO_2_), chromium oxide (Cr_2_O_3_),silicon nitride (Si_3_N_4_) and aluminum oxide (Al_2_O_3_) were purchased from Daisy Impex Chemicals (Average particle size = 0.2–5 μm; New Delhi, India). 

**Figure 1:**
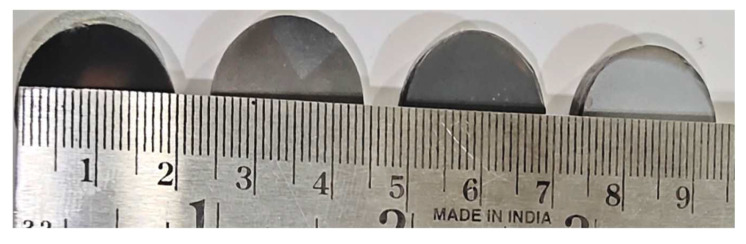
Manufactured composite samples.

The spark plasma sintering (SPS) procedure was used to create samples with varying weight percentages of selected ceramic materials, as shown in Table 1. First, the selected ceramic materials were milled for 4 h at 300 rpm in a toluene solution (50 mL) using a grinding medium (tungsten). The mixture of powders was separated from the solution by filtration through fine filter paper, and then oven dried. Then, in a vacuum, samples sized 10 mm (in thickness) and 20 mm (in diameter) were sintered using SPS-725 equipment from DR. SINTER, Japan. The sets of prepared composite powder mixtures were carefully placed into a 20 mm diameter graphite die. A sheet of graphitic paper was placed between the punch and the powders and between the die and the powders for easy removal of the sintered sample. A pressure of 60 MPa was applied throughout the sintering cycle. The sintering temperature was increased to 1400 °C at a rate of 300 °C/min [40]. A 3 min holding time was used when the maximum temperature was reached. The polished samples (Figure 1) were then subjected to physical, mechanical, and wear tests.

### 2.2. Measurements

The experimental density was calculated using Wensar density measurement equipment, whereas the mixture rule was used for void content determination. The nanoindentation test was carried out as per ASTM E2546 standard to determine Young’s modulus and hardness properties of manufactured hip implant composites. For this, a Hysitron TI 750-D (Ubi-1 model) testing machine was used. The Berkovich diamond indenter was used with a 150 nm tip radius. The indentation time was 10 s, and the applied load was 5000 µN with a 500 µN/s loading rate [41]. The load and associated penetration depth values were recorded. After that, Young’s modulus and hardness were computed with the help of Pharr and Oliver’s model [42]. On each composite sample, fifteen indents were made to increase the reliability of the data. The compression tests performed on a universal testing machine (Model No. 8862, INSTRON, High Wycombe, UK) on the manufactured composite samples with 1 mm/min cross-head speed. Fracture toughness of manufactured hip implant composites was computed by measuring the crack formed due to the Vickers indentation and half-penny crack system. Therefore, the manufactured composites were penetrated with the help of a hardness tester from Buehler, USA, for crack generation. The penetration time was 13 s, and the applied load was 20 N. The length of the generated crack was counted using a scanning electron microscope. Finally, the Anstis model was implemented to compute the fracture toughness ($K_{IC}$) as [43];

(1) $$K_{IC}\;=\;0.016\;{\left(\frac{Y}{H}\right)}^{\frac{1}{2}}\;\times \;\frac{f}{c^{\frac{3}{2}}}$$

where, *H* = Hardness, *Y* = Young’s modulus, *f* = Applied load; c = a + l, l = crack length, *a* = half diagonal of the indent.

To increase the accuracy of the obtained results, the experiments were repeated five times and the mean data were reported with the estimated error.

### 2.3. Wear Characterization

**Figure 2:**
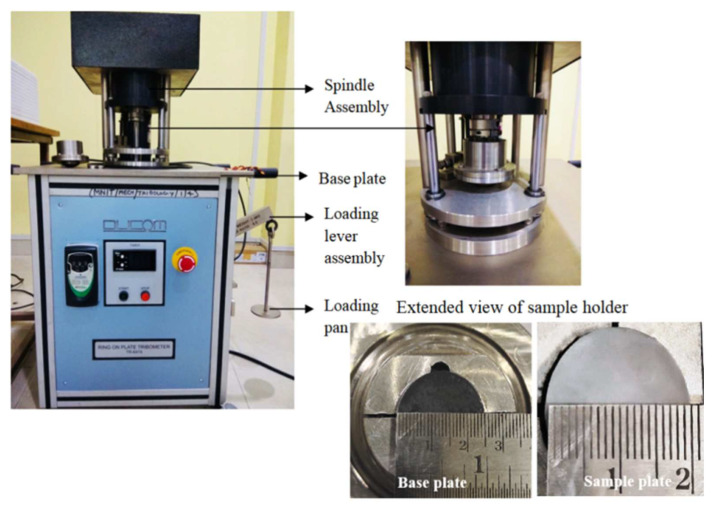
Ring on plate tribometer.

The manufactured hip implant composites were evaluated for wear behaviour on a ring-on-plate type tribometer (Ducom, India; model: TR-6474). The systematic of the machine is presented in Figure 2. The wear tests were conducted according to ISO 6474-1:2010 [44]. The tribometer’s structure is built of mild steel. The structure’s upper part is linked to a base plate. A support stand is welded to the base plate, and a crank mechanism is installed. A motor is mounted vertically on a welded support stand. The motor shaft is usually visible above the stand. To pivot alongside the shaft, a crank wheel is fixed to it. The wheel rotates the axle via a switch, oscillating the composite sample plate by 25 degrees while the base plate remains stationary. Sintered and polished composite samples were placed on the top plate and rotated at the selected frequency across an arc of 25° against a base plate. The base plate with dimensions of 25 mm (diameter) × 6 mm (height) was selected from the manufactured composites. The base plate was manufactured using the same sintering procedure described in Section 2.1. Wear tests were carried out with a frequency of 50 Hz and 500 N load for one million cycles using simulated body fluid conditions as per ISO 23317 [45]. After the completion of the test, wear was computed on a volume basis according to Equation (2). For each composite, three samples were used for wear estimation, and average is reported.

(2) $$Κ\;=\;\frac{w_{2}\;-\;w_{1}}{\rho }$$

Here, $w_{1}$ = sample weight before test, $w_{2}$ = sample weight after test, ρ = sample density.

### 2.4. Determination and Implication of Criterion

The evaluated physical (density, void content), mechanical (hardness, elastic modulus, fracture toughness, and compressive strength) and wear properties were taken as a criterion in the ranking process of the developed hip implant composites. The implications of the selected criterion are given as:

Criterion-1 (C-1): Density (g/cm^3^, Lower-is-better)
Criterion-2 (C-2): Void content (Volume-%, Lower-is-better)
Criterion-3 (C-3): Hardness (GPa, Higher-is-better)
Criterion-4 (C-4): Indentation depth (nm, Lower-is-better)
Criterion-5 (C-5): Elastic modulus (GPa, Higher-is-better)
Criterion-6 (C-6): Fracture toughness (MPa.m^1/2^, Higher-is-better)
Criterion-7 (C-7): Compressive strength (GPa, Higher-is-better)
Criterion-8 (C-8): Wear (mm^3^/million cycles, Lower-is-better)

### 2.5. Evaluation Methodology

**Figure 3:**
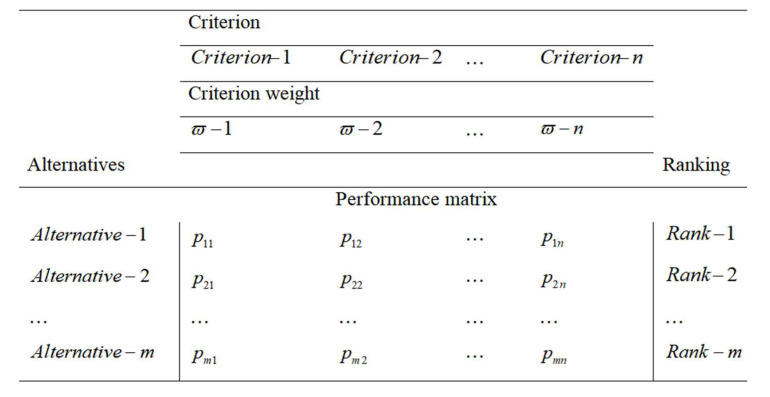
A MCDM model.

**Figure 4:**
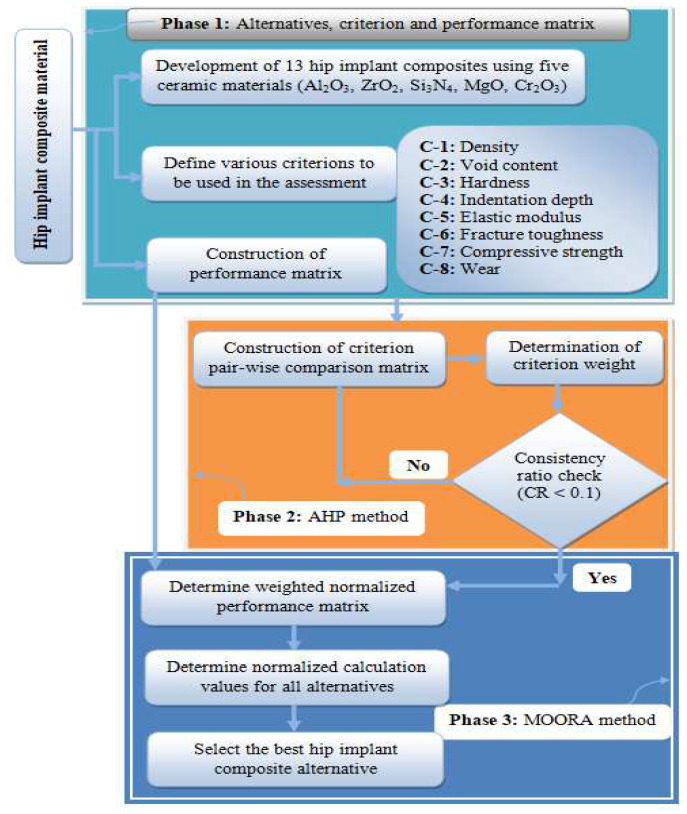
Architecture of the hybrid AHP-MOORA approach.

The MCDM approaches are generally used to rank a predetermined number of alternatives considering a set of criteria. The various parts, namely criterion, alternatives, and performance matrix, of any MCDM approach are presented in Figure 3. The algorithm of this hybrid AHP-MOORA approach is systematically presented in Figure 4. 

The proposed hybrid AHP-MOORA approach contains three phases, namely:

Phase 1: Alternatives, criterion and creation of performance matrix
Phase 2: AHP for criterion weight determination
Phase 3: MOORA approach for alternatives ranking

#### 2.5.1. Phase 1: Alternatives, Criterion and Creation of Performance Matrix

In order to initiate any MCDM approach, the number of criteria and alternatives needs to be defined first. For selected alternatives (presented in Table 1) and criteria (described in Section 2.4), a performance matrix is generated as:

(3) $$P_{m\;\times \;n}\;=\;\begin{array}{c} A_{1}\\ A_{i}\\ \vdots \\ A_{m} \end{array}\overset{\begin{array}{llll} C_{1}\;\;\;\;\;\;\;\;\;\;\;\;\;\;\; & C_{j} & \;\;\;\;\;\;\;\;\;\cdots \;\;\;\;\;\;\;\; & \;\;\;C_{n} \end{array}\;\;\;}{\left[\begin{array}{cccc} p_{11} & p_{1j} & \cdots \cdots \cdots & p_{1n}\\ p_{i1} & p_{ij} & \cdots \cdots \cdots & p_{in}\\ \vdots & \vdots & \ddots & \vdots \\ p_{m1} & p_{mj} & \cdots \cdots \cdots & p_{mn} \end{array}\right]}$$

where, the factor $p_{ij}$ symbolizes the value of $i^{th}$ alternative with respect to $j^{th}$ criterion.

#### 2.5.2. Phase 2: AHP for Criteria Weight Determination

For weight estimation, a pair-wise comparison matrix first structured using scale of 1 to 9 [46]. For *n* criteria $\left\{C\;-\;j;\;j\;=\;1,\;\;2,\cdots ,\;\;n\right\}$, the structured matrix (ℏ) is:

(4) $$\hbar_{n\;\times \;n}\;=\;\begin{array}{l} C_{1}\\ C_{k}\\ \vdots \\ C_{n} \end{array}\overset{\begin{array}{llll} C_{1}\;\;\;\;\;\;\;\;\;\;\;\;\;\;\; & C_{j} & \;\;\;\;\;\;\;\;\;\cdots \;\;\;\;\;\;\;\; & \;\;\;C_{n} \end{array}\;\;\;}{\left[\begin{array}{cccc} h_{11} & h_{1j} & \cdots \cdots \cdots & h_{1n}\\ h_{k1} & h_{kj} & \cdots \cdots \cdots & h_{kn}\\ \vdots & \vdots & \ddots & \vdots \\ h_{n1} & h_{nj} & \cdots \cdots \cdots & h_{nn} \end{array}\right]}$$

where, $h_{kj}$ is the comparative importance of $k^{th}$ to $j^{th}$ criteria. Diagonally the criteria values are self-compared, hence $h_{kj}\;=\;1$, where *k* = *j*; *k*, *j* = 1, 2…*n*.

By using geometric mean method, the criteria weight is computed as:

(5) $$\varpi_{i}\;=\;\frac{{\left(\prod_{j\;=\;1}^{n}h_{kj}\right)}^{\frac{1}{n}}}{\sum_{i\;=\;1}^{n}{\left(\prod_{j\;=\;1}^{n}h_{kj}\right)}^{\frac{1}{n}}}$$

Finally, to ensure the stability of weight determined, consistency ratio (CR) is determined as:

(6) $$CR\;=\;\frac{\lambda_{\max}\;-\;n}{RI\;\times \;(n\;-\;1)}$$

Here, RI (random index) is the order value of the matrix [47] and $\lambda_{\max}$ is the maximum Eigen value determined using the following equation:

(7) $$\hbar_{n\times n}\times \varpi_{i}\;=\;\lambda_{\max}\times \varpi_{i}$$

#### 2.5.3. Phase 3: MOORA Approach for Alternatives Ranking

Proposed by Brauers [48], the MOORA approach has successfully been applied in various decision-making problems [49,50]. In MOORA methodology, the constructed performance matrix is first normalized in between 0–1 by using the following equation:

(8) $$p_{ij}^{*}\;=\;\frac{p_{ij}}{\sum_{i\;=\;1}^{m}p_{ij}^{2}}$$

Next, the weighted normalized performance matrix was constructed as:

(9) Ν=ijpij∗ × ϖj

Finally, the normalized values were added for favourable (higher-is-better) criteria and subtracted for non-favourable (lower-is-better) criteria as:

(10) Ψi = Ψ−1Ψ2

(11) Ψ1 = ∑j = 1αΝij

(12) Ψ2 = ∑j = α + 1nΝij

where $\Psi_{i}$ represents the computation of *i^th^* alternative. The α and *n*−α values represent the favourable and non-favourable criteria. The final ranking of the alternatives is established by sorting the $\Psi_{i}$ values in descending order.

## 3. Results and Discussion

### 3.1. Criteria Interpretation 

The results of evaluated wear and physicomechanical properties are presented in Table 2. The density of the manufactured composites remains in the range of 3.92 g/cm^3^ to 4.307 g/cm^3^. The alternative without ZrO_2_ content, i.e., A-11, had the lowest density, and alternative A-13 with 30 wt.% ZrO_2_ was highest. The alternative A-11 with 10 wt.% ZrO_2_ content exhibits a density of 4.048 g/cm^3^, whereas for alternatives A-1 to A-10 with 20 wt.% ZrO_2_ the densities fluctuate in between 4.166 ± 0.024 g/cm^3^. This trend in density may be ascribed to the presence of high dense ZrO_2_ content. This increase in density with increased ZrO_2_ content is correlated to its lower sintering temperatures in relation to Al_2_O_3_ content [51]. The lowest and highest void contents were observed for A-1 and A-5 alternatives, respectively. The void content of alternative A-5 without MgO added composite remains highest (0.0089%), whereas, for MgO based composites, it remains lower and fluctuates between 0.0021% and 0.0063%. It was accounted for that in a higher temperature sintering process, MgO transforms into liquids and attempts to fill the pores resulted in reduced void content [17]. The alternative A-12 had 83 wt.% Al_2_O_3_ content showed a minor indentation depth (63.25 nm) with the highest hardness values (28.81 GPa). The observed value of hardness was nearly 50% higher, and indentation depth was almost 28% lower than the values observed for alternatives A-1, A-5 and A-8, where they remain 19.32 ± 0.13 GPa and 81 ± 5 nm, respectively.

In the literature, the hardness of 18.3 GPa for MgO doped Ce-TZP/Al_2_O_3_, 18.70 ± 6.63 GPa for Si_3_N_4_-MgO, 19.6 ± 0.2 GPa for graphene oxide filled 3Y-ZrO_2_, and 28±2 GPa for zirconia-toughened alumina-based ceramic composites were reported by Zhang et al. [17], Mazzocchi et al. [52], Zhang et al. [53] and Bull et al. [54], respectively. Comparatively, the highest hardness of 28.81 GPa with a minor indentation depth of 63.25 nm remains much higher than the results of Zhang et al. [17], Mazzocchi et al. [52] and Zhang et al. [53] and remains nearly the same as the results of Bull et al. [54]. The elastic modulus, fracture toughness, and compressive strength for the alternative A-3 remained 276.55 GPa, 11.41 MPa.m^1/2^ and 2.841 GPa. With an increase in Al_2_O_3_ content to 73 wt.% and a corresponding decrease in Si_3_N_4_ content to 2.5 wt.%, a small increment in elastic modulus, fracture toughness and compressive strength was observed for alternative A-9. Further increase in Al_2_O_3_ content to 83 wt.% with a corresponding decrease in ZrO_2_ content to 10 wt.%, an increment of ~1–4% in elastic modulus (291 GPa), fracture toughness (11.97 MPa.m^1/2^), and compressive strength (2.894 GPa) was registered and remained maximum for alternative A-12. Similar results of elastic modulus and fracture toughness was reported by Zhang et al. [53] and Bull et al. [54] for graphene oxide filled 3Y-ZrO_2_ and zirconia-toughened alumina-based ceramic composites. While studying the influence of MgO (0 to 0.3 wt.%) and Ce-TZP (5 to 25 vol.%) in MgO doped Ce-TZP/Al_2_O_3_ bioceramic-based composites, Zhang and co-workers [17] reported similar results for fracture toughness. As per [17], a significant improvement in fracture toughness was reported with increasing Ce-TZP concentration and remains maximum (12.10 MPa.m^1/2^) with 20 vol.% of Ce-TZP and 0.2 wt.% of MgO content. Recently, Sedlák et al. [55] investigated the nanomechanical properties of spark plasma sintered (1400 °C) oxide ceramics (70 wt.% Al_2_O_3_ + 30 wt.% ZrO_2_) composites using nanoindentation. A Berkovich diamond indenter with a continuous stiffness measuring mode with a strain rate of 0.05 s^−1^ and 200 nm depth was used. The authors claimed a hardness of 29.5 GPa, elastic modulus of 337 GPa, and fracture toughness of 3.72 MPa.m^1/2^. The highest experimental values of hardness and elastic modulus in the present work are close to the results of Sedlák et al. [55], whereas the highest fracture toughness was almost 3.2 times higher than the results reported in ref. [55]. 

The Al_2_O_3_–ZrO_2_ are widely known as mechanically and biologically compatible ceramic materials [42]. The performance of the final composite depends upon the composition, and it was reported that the uniform distribution of ZrO_2_ particles was greatly hampered in the Al_2_O_3_-based composites with their increased (>20 wt.%) concentration. These structural inhomogeneities could form large voids in the composite. A higher temperature is required to eliminate these voids, which could cause the unusual grain growth of Al_2_O_3_, having a detrimental impact on the mechanical and wear performance of the composites [56]. The inclusion of various ceramics is reported to alter the performance of Al_2_O_3_–ZrO_2_ ceramic-based composites beneficially. It was reported that the addition of MgO resists the growth of Al_2_O_3_ grains due to the microstructure pinning effect [57,58]. The addition of Cr_2_O_3_ was reported to enhance the mechanical performance of Al_2_O_3_ as it increases crack bridging [59]. Furthermore, covalently bonded Si_3_N_4_ has much higher mechanical strength than well-known oxide bioceramics and has been reported to enhance the strength and wear performance of Al_2_O_3_–ZrO_2_ based composites [60]. The wear remains 0.0078 mm^3^/million cycles for alternative A-3 having 70.50 wt.% and 5 wt.% Al_2_O_3_ and Si_3_N_4_. A reduction of nearly 3% in wear was reported for alternative A-9 by increasing Al_2_O_3_ content to 73 wt.% and decreasing Si_3_N_4_ content to 2.5 wt.%. Further reduction in wear was observed for alternative A-12 (0.0071 mm^3^/million cycles) by increasing Al_2_O_3_ content to 83 wt.% and decreasing ZrO_2_ content to 10 wt.%. The observed trends for the wear rate of manufactured composites were consistent with the reported range of implant composites. In the literature, the wear rate of 0.01–0.1 mm^3^ per million cycles was reported for alumina-on-alumina implants by Gallo et al. [61], 0.0036 mm^3^ per million cycles for polycrystalline diamond-based materials by Harding et al. [62] and 0.005 mm^3^ per million cycles for nanocrystalline diamond-coated ceramic hip joints by Amaral and co-workers [63]. The lowest wear rate of 0.0071 mm^3^ per million-cycles for alternative A-12 remained much lower than the results of alumina-on-alumina implants [61] but remained nearly 30 to 49% higher than the results of polycrystalline diamond [62] and nanocrystalline diamond-coated implants [63].

The assessed criteria play a decisive role in designing successful hip implant materials. Lower experimental values are desirable for many criteria like density, void content, and wear, whereas higher values are desirable for hardness, indentation depth, elastic modulus, fracture toughness, and compressive strength. As shown in Table 3, no alternative can yield preferred lower and higher values for all criteria at a single time. Accordingly, to pick the best choice by considering all the criteria together, a hybrid AHP-MOORA approach was implemented.

### 3.2. Ranking of Alternative

#### 3.2.1. Weight Calculation

The weights of the selected criteria were determined using the AHP approach. To begin, the criteria were compared using a pair-wise matrix, as shown in Table 3. The results of the AHP technique are shown in Table 4. The criteria weight order is the following: C-8 (0.340) > C-6 (0.227) > C-5 (0.111) > C-3 (0.108) > C-7 (0.074) > C-4 (0.062) > C-1 (0.048) > C-2 (0.030). Moreover, the CR value was determined as 0.023, which is lower than the prescribed limit of 0.1. Therefore, the computed weights were consistent and further utilised to rank the alternatives.

#### 3.2.2. Ranking Analysis

**Figure 5:**
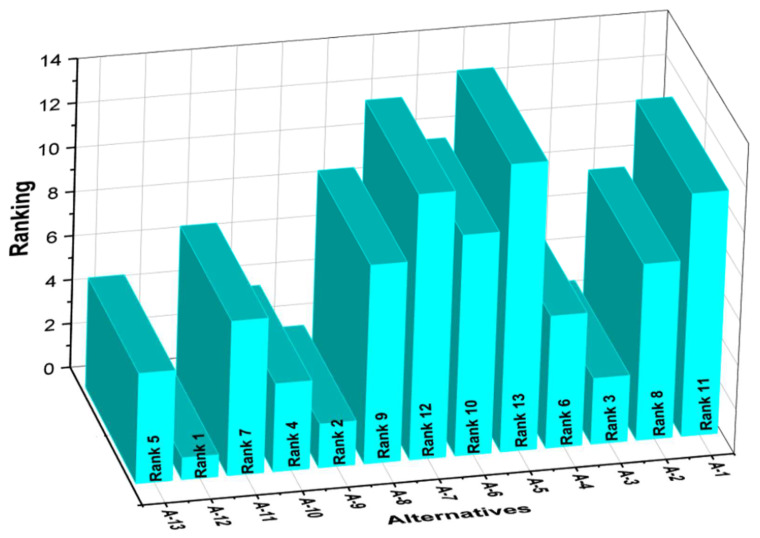
Ranking of hip implant alternatives.

Table 2 shows the experimental results of the researched hip implant composites, which were used as a performance matrix. Because each alternative produces a different result for each criterion, the performance matrix values were normalized in the range of 0–1 using Equation (8). Table 5 displays the normalized performance matrix. Following normalization, a weighted normalized matrix is created using Equation (9) and shown in Table 6. After weighted normalization, the value of each alternative is determined using Equations (10)–(12) and presented in Table 6. The value of the composite A-12 is the highest (0.1187), indicating that it is the best of all the possible options. The composites A-9 (0.1111) and A-3 (0.1111) come in second and third, respectively (0.1042). The composite A-5 demonstrates the least preference with a value of -0.0834. The ranking results are summarized in Table 6 and illustrated in Figure 5. Overall, the hip implant composites ranking in downward order is A-12, A-9, A-3, A-10, A-13, A-4, A-11, A-2, A-8, A-6, A-1, A-7 and A-5. This investigation revealed that the composite A-12, which contains 73 weight percent Al_2_O_3_, 20 weight percent ZrO_2_, 2.5 weight percent Si_3_N_4_, 2.5 weight percent Cr_2_O_3_, and 3 weight percent MgO had the best combination of wear and physicomechanical properties.

### 3.3. Sensitivity Analysis 

Furthermore, a sensitivity analysis was conducted to assess the ranking findings’ robustness and stability in relation to the chosen criterion weight. This sensitivity analysis has been carried out by changing the weight of the two critical criteria, namely fracture toughness (C-6) and wear (C-8), the most weighted criteria with weights of 0.227 and 0.340, respectively, among the selected eight criteria. The sensitivity analysis was executed by increasing and decreasing a criterion weight in three steps (±5%, ±10% and ±15%). The remaining seven criterion weights were adjusted proportionately to maintain the sum of all criterion weights equal to one. Table 7 and Table 8 show the ranking of produced ceramic hip implant composites based on changes in the weight of selected criteria C-6 and C-8. 

The variation of C-6 and C-8 weights caused a little sensitivity in the composite ranking. However, within the range of ±5%, ±10% and ±15%, except for A-4 and A-11, all other composite alternatives retain their respective rankings irrespective of the weightage of the C-6 and C-8. As the weightage of C-6 is increased to +10% and +15%, the ranking of the alternative A-4 goes down by one place, and the ranking of A-11 goes up by one place. Similarly, as the weightage of C-8 is decreased to −10% and −15%, the ranking of the alternative A-4 goes up by one place, and the ranking of A-11 goes down by one place. Such variation in ranking may be attributed to the fact that the fracture toughness of A-4 is inferior compared to A-11, whereas the wear performance of A-4 is superior compared to A-11. Hence, as the weightage of C-6 is increased by +10% and +15%, the ranking of A-4 goes up by one, and as the weightage of C-8 is decreased by −10% and −15%, the ranking of A-11 loses its position by one in the hierarchy of hip implant composites.

## 4. Conclusions

This study proposed a hybrid multicriteria decision-making framework to solve the problem of selecting the best hip implant composite satisfying a maximum number of preset performance criteria. Thirteen hip implant composites based on a varying proportion of various ceramics (magnesium oxide, zirconium oxide, chromium oxide, silicon nitride and aluminium oxide) were developed and tested for wear and physicomechanical properties. The experimental results of density, void content, hardness, indentation depth, elastic modulus, fracture toughness, compressive strength, and wear were considered as performance criteria in the assessment. With the introduction of AHP method, the weight for each criterion quantified from higher to the lower order as wear (0.340) > fracture toughness (0.227) > elastic modulus (0.111) > hardness (0.108) > compressive strength (0.074) > indentation depth (0.062) > density (0.048) > void content (0.030). By applying MOORA approach, the performance order of hip implant composites could be obtained as A-12 > A-9 > A-3 > A-10 > A-13 > A-4 > A-11 > A-2 > A-8 > A-6 > A-1 > A-7 > A-5. The hip implant alternative A-12 having 83 wt.% of aluminium oxide, 10 wt.% of zirconium oxide, 2.5 wt.% of silicon nitride, 1.5 wt.% of chromium oxide and 3 wt.% of magnesium oxide exhibits optimal wear and physicomechanical properties. Sensitivity analysis shows that a change in criterion weight did not significantly change the overall ranking of the hip implant composite, further enhancing the reliability of the results. The application shows that the proposed AHP-MOORA approach may be used as a trustworthy decision-making framework in selecting hip implant materials along with various decision-making situations and research domains.

## Figures and Tables

**Table 1:** Compositional detail of hip implant composite alternatives.

Composition (wt.%)	Alternatives												
	A-1	A-2	A-3	A-4	A-5	A-6	A-7	A-8	A-9	A-10	A-11	A-12	A-13
Al_2_O_3_	72	71.25	70.5	69.75	73.5	72	69	75.5	73	68	93	83	63
ZrO_2_	20	20	20	20	20	20	20	20	20	20	0	10	30
Si_3_N_4_	5	5	5	5	5	5	5	0	2.5	7.5	2.5	2.5	2.5
MgO	3	3	3	3	0	1.5	4.5	3	3	3	3	3	3
Cr_2_O_3_	0	0.75	1.5	2.25	1.5	1.5	1.5	1.5	1.5	1.5	1.5	1.5	1.5

**Table 2:** Experimental results.

Alternatives	C-1	C-2	C-3	C-4	C-5	C-6	C-7	C-8
A-1	4.151 ± 0.001	0.0021	19.19 ± 0.68	79.03 ± 2.32	226.12 ± 6.85	04.19 ± 0.17	2.715 ± 0.07	0.0246 ± 0.0008
A-2	4.154 ± 0.002	0.0033	21.34 ± 1.07	72.92 ± 1.82	239.10 ± 6.29	04.85 ± 0.24	2.772 ± 0.07	0.0152 ± 0.0005
A-3	4.158 ± 0.002	0.0042	27.83 ± 1.16	69.61 ± 1.55	276.55 ± 9.88	11.41 ± 0.41	2.841 ± 0.07	0.0078 ± 0.0004
A-4	4.161 ± 0.001	0.0054	20.93 ± 0.88	73.68 ± 1.67	232.05 ± 5.53	04.90 ± 0.15	2.796 ± 0.07	0.0127 ± 0.0006
A-5	4.152 ± 0.001	0.0089	19.33 ± 0.76	86.03 ± 2.26	243.12 ± 6.57	05.75 ± 0.19	2.730 ± 0.08	0.0323 ± 0.0010
A-6	4.156 ± 0.002	0.0063	20.99 ± 0.84	82.92 ± 1.68	244.10 ± 4.88	07.84 ± 0.27	2.793 ± 0.04	0.0276 ± 0.0008
A-7	4.150 ± 0.002	0.0045	19.93 ± 0.72	87.68 ± 1.58	229.05 ± 4.16	05.87 ± 0.23	2.816 ± 0.03	0.0289 ± 0.0008
A-8	4.190 ± 0.001	0.0044	19.45 ± 0.61	76.14 ± 2.82	235.85 ± 5.24	08.63 ± 0.26	2.718 ± 0.05	0.0213 ± 0.0009
A-9	4.176 ± 0.002	0.0038	28.64 ± 1.10	67.93 ± 1.66	280.18 ± 6.83	11.84 ± 0.48	2.861 ± 0.05	0.0076 ± 0.0003
A-10	4.142 ± 0.003	0.0043	22.69 ± 0.71	72.18 ± 2.01	245.12 ± 4.71	09.93 ± 0.34	2.810 ± 0.06	0.0139 ± 0.0007
A-11	3.920 ± 0.002	0.0045	19.93 ± 0.22	79.74 ± 2.28	263.12 ± 7.97	08.47 ± 0.22	2.814 ± 0.06	0.0196 ± 0.0006
A-12	4.048 ± 0.002	0.0032	28.81 ± 0.96	63.25 ± 1.64	291.00 ± 9.72	11.97 ± 0.28	2.894 ± 0.06	0.0071 ± 0.0002
A-13	4.307 ± 0.002	0.0058	20.79 ± 0.72	78.83 ± 1.43	253.05 ± 7.23	07.45 ± 0.21	2.818 ± 0.05	0.0127 ± 0.0003

**Table 3:** Pair-wise matrix for weight determination.

	C-1	C-2	C-3	C-4	C-5	C-6	C-7	C-8
C-1	1	1.5	0.5	0.67	0.33	0.25	0.67	0.17
C-2	0.67	1	0.25	0.5	0.2	0.17	0.33	0.13
C-3	2	4	1	1.5	2	0.33	1.50	0.25
C-4	1.5	2	0.67	1	0.67	0.25	0.5	0.2
C-5	3	5	0.5	1.5	1	0.5	2	0.33
C-6	4	6	3	4	2	1	4	0.5
C-7	1.50	3	0.67	2	0.5	0.25	1	0.2
C-8	6	8	4	5	3	2	5	1

**Table 4:** Results of AHP method.

Criterion	$\mathrm{Weight}\;(\varpi_{i})$	Consistency Parameters	
C-1	0.048		
C-2	0.030		
C-3	0.108	λ_max_ = 8.23	
C-4	0.062	CI = 0.033	CR = 0.023
C-5	0.111	RI = 1.41	
C-6	0.227		
C-7	0.074		
C-8	0.340		

**Table 5:** Normalized performance matrix.

Alternatives	C-1	C-2	C-3	C-4	C-5	C-6	C-7	C-8
A-1	0.2778	0.1167	0.2358	0.2867	0.2494	0.1390	0.2691	0.3465
A-2	0.2780	0.1833	0.2623	0.2645	0.2637	0.1609	0.2747	0.2141
A-3	0.2783	0.2333	0.3420	0.2525	0.3050	0.3786	0.2815	0.1099
A-4	0.2785	0.3000	0.2572	0.2673	0.2560	0.1626	0.2771	0.1789
A-5	0.2779	0.4944	0.2376	0.3121	0.2682	0.1908	0.2705	0.4549
A-6	0.2781	0.3500	0.2580	0.3008	0.2692	0.2602	0.2768	0.3887
A-7	0.2777	0.2500	0.2449	0.3181	0.2526	0.1948	0.2791	0.4070
A-8	0.2804	0.2444	0.2390	0.2762	0.2601	0.2864	0.2693	0.3000
A-9	0.2795	0.2111	0.3520	0.2464	0.3090	0.3929	0.2835	0.1070
A-10	0.2772	0.2389	0.2789	0.2618	0.2704	0.3295	0.2785	0.1958
A-11	0.2623	0.2500	0.2449	0.2893	0.2902	0.2811	0.2789	0.2761
A-12	0.2709	0.1778	0.3541	0.2294	0.3210	0.3972	0.2868	0.1000
A-13	0.2882	0.3222	0.2555	0.2860	0.2791	0.2472	0.2793	0.1789

**Table 6:** Weighted matrix and ranking of alternatives.

	Weighted Matrix								Ranking of Alternatives			
Alternatives	C-1	C-2	C-3	C-4	C-5	C-6	C-7	C-8	$\Psi_{1}$	$\Psi_{2}$	$\Psi_{i}$	Ranking
A-1	0.0133	0.0035	0.0255	0.0178	0.0277	0.0316	0.0199	0.1178	0.1046	0.1524	−0.0478	11
A-2	0.0133	0.0055	0.0283	0.0164	0.0293	0.0365	0.0203	0.0728	0.1145	0.1080	0.0064	8
A-3	0.0134	0.0070	0.0369	0.0157	0.0339	0.0860	0.0208	0.0374	0.1776	0.0734	0.1042	3
A-4	0.0134	0.0090	0.0278	0.0166	0.0284	0.0369	0.0205	0.0608	0.1136	0.0998	0.0139	6
A-5	0.0133	0.0148	0.0257	0.0193	0.0298	0.0433	0.0200	0.1547	0.1188	0.2022	−0.0834	13
A-6	0.0134	0.0105	0.0279	0.0186	0.0299	0.0591	0.0205	0.1322	0.1373	0.1747	−0.0374	10
A-7	0.0133	0.0075	0.0265	0.0197	0.0280	0.0442	0.0207	0.1384	0.1194	0.1789	−0.0596	12
A-8	0.0135	0.0073	0.0258	0.0171	0.0289	0.0650	0.0199	0.1020	0.1396	0.1399	−0.0003	9
A-9	0.0134	0.0063	0.0380	0.0153	0.0343	0.0892	0.0210	0.0364	0.1825	0.0714	0.1111	2
A-10	0.0133	0.0072	0.0301	0.0162	0.0300	0.0748	0.0206	0.0666	0.1555	0.1033	0.0523	4
A-11	0.0126	0.0075	0.0265	0.0179	0.0322	0.0638	0.0206	0.0939	0.1431	0.1319	0.0112	7
A-12	0.0130	0.0053	0.0382	0.0142	0.0356	0.0902	0.0212	0.0340	0.1853	0.0666	0.1187	1
A-13	0.0138	0.0097	0.0276	0.0177	0.0310	0.0561	0.0207	0.0608	0.1354	0.1020	0.0333	5

**Table 7:** Weight sensitivity of C-6 (fracture toughness) on ranking.

Alternatives	Weight Level						
	−15%	−10%	−5%	Original	+5%	+10%	+15%
	$\Psi_{i}\mathrm{Value}\;(\mathrm{Ranking})$						
A-1	−0.0538 (11)	−0.0517 (11)	−0.0497 (11)	−0.0477 (11)	−0.0457 (11)	−0.0437 (11)	−0.0416 (11)
A-2	0.0003 (8)	0.0024 (8)	0.0045 (8)	0.0065 (8)	0.0086 (8)	0.0106 (8)	0.0127 (8)
A-3	0.0917 (3)	0.0959 (3)	0.1001 (3)	0.1043 (3)	0.1086 (3)	0.1128 (3)	0.1170 (3)
A-4	0.0072 (6)	0.0095 (6)	0.0117 (6)	0.0139 (6)	0.0162 (6)	0.0184 (7)	0.0206 (7)
A-5	−0.0936 (13)	−0.0901 (13)	−0.0867 (13)	−0.0833 (13)	−0.0800 (13)	−0.0766 (13)	−0.0731 (13)
A-6	−0.0487 (10)	−0.0448 (10)	−0.0411 (10)	−0.0373 (10)	−0.0335 (10)	−0.0297 (10)	−0.0259 (10)
A-7	−0.0685 (12)	−0.0654 (12)	−0.0625 (12)	−0.0595 (12)	−0.0565 (12)	−0.0535 (12)	−0.0505 (12)
A-8	−0.0116 (9)	−0.0078 (9)	−0.0040 (9)	−0.0002 (9)	0.0036 (9)	0.0074 (9)	0.0112 (9)
A-9	0.0983 (2)	0.1026 (2)	0.1069 (2)	0.1112 (2)	0.1155 (2)	0.1198 (2)	0.1241 (2)
A-10	0.0404 (4)	0.0444 (4)	0.0484 (4)	0.0524 (4)	0.0564 (4)	0.0603 (4)	0.0643 (4)
A-11	0.0004 (7)	0.0041 (7)	0.0077 (7)	0.0113 (7)	0.0149 (7)	0.0185 (6)	0.0222 (6)
A-12	0.1051 (1)	0.1093 (1)	0.1135 (1)	0.1178 (1)	0.1220 (1)	0.1262 (1)	0.1305 (1)
A-13	0.0237 (5)	0.0270 (5)	0.0302 (5)	0.0334 (5)	0.0366 (5)	0.0399 (5)	0.0431 (5)

**Table 8:** Weight sensitivity of C-8 (wear) on ranking.

Alternatives	Weight Level						
	−15%	−10%	−5%	Original	+5%	+10%	+15%
	$\Psi_{i}\mathrm{Value}\;(\mathrm{Ranking})$						
A-1	−0.0285 (11)	−0.0348 (11)	−0.0414 (11)	−0.0477 (11)	−0.0540 (11)	−0.0614 (11)	−0.0669 (11)
A-2	0.0191 (8)	0.0150 (8)	0.0106 (8)	0.0065 (8)	0.0024 (8)	−0.0028 (8)	−0.0060 (8)
A-3	0.1138 (3)	0.1108 (3)	0.1073 (3)	0.1043 (3)	0.1014 (3)	0.0971 (3)	0.0949 (3)
A-4	0.0238 (7)	0.0206 (7)	0.0172 (6)	0.0139 (6)	0.0107 (6)	0.0065 (6)	0.0041 (6)
A-5	−0.0610 (13)	−0.0685 (13)	−0.0758 (13)	−0.0833 (13)	−0.0908 (13)	−0.0991 (13)	−0.1057 (13)
A-6	−0.0165 (10)	−0.0234 (10)	−0.0304 (10)	−0.0373 (10)	−0.0442 (10)	−0.0520 (10)	−0.0581 (10)
A-7	−0.0378 (12)	−0.0450 (12)	−0.0523 (12)	−0.0595 (12)	−0.0667 (12)	−0.0748 (12)	−0.0811 (12)
A-8	0.0169 (9)	0.0113 (9)	0.0054 (9)	−0.0002 (9)	−0.0058 (9)	−0.0125 (9)	−0.0173 (9)
A-9	0.1209 (2)	0.1178 (2)	0.1142 (2)	0.1112 (2)	0.1082 (2)	0.1037 (2)	0.1015 (2)
A-10	0.0650 (4)	0.0609 (4)	0.0565 (4)	0.0524 (4)	0.0483 (4)	0.0430 (4)	0.0397 (4)
A-11	0.0275 (6)	0.0222 (6)	0.0166 (7)	0.0113 (7)	0.0060 (7)	−0.0003 (7)	−0.0048 (7)
A-12	0.1276 (1)	0.1245 (1)	0.1208 (1)	0.1178 (1)	0.1147 (1)	0.1102 (1)	0.1080 (1)
A-13	0.0437 (5)	0.0403 (5)	0.0368 (5)	0.0334 (5)	0.0300 (5)	0.0256 (5)	0.0231 (5)

## Data Availability

The data that support the findings of this study are available from the first author (T.S.) upon reasonable request.
